# Cytotoxicity-Related Bioeffects Induced by Nanoparticles: The Role of Surface Chemistry

**DOI:** 10.3389/fbioe.2019.00414

**Published:** 2019-12-12

**Authors:** Hainan Sun, Cuijuan Jiang, Ling Wu, Xue Bai, Shumei Zhai

**Affiliations:** ^1^Key Laboratory of Colloid and Interface Chemistry of the Ministry of Education, School of Chemistry and Chemical Engineering, Shandong University, Jinan, China; ^2^Shandong Vocational College of Light Industry, Zibo, China; ^3^School of Environmental Science and Engineering, Shandong University, Qingdao, China

**Keywords:** nanoparticles, surface chemistry, charge, hydrophobicity, PEG, cytotoxicity

## Abstract

Nanoparticles (NPs) are widely used in a variety of fields, including those related to consumer products, architecture, energy, and biomedicine. Once they enter the human body, NPs contact proteins in the blood and interact with cells in organs, which may induce cytotoxicity. Among the various factors of NP surface chemistry, surface charges, hydrophobicity levels and combinatorial decorations are found to play key roles inregulating typical cytotoxicity-related bioeffects, including protein binding, cellular uptake, oxidative stress, autophagy, inflammation, and apoptosis. In this review, we summarize the recent progress made in directing the levels and molecular pathways of these cytotoxicity-related effects by the purposeful design of NP surface charge, hydrophobicity, and combinatorial decorations.

## Introduction

Due to their unique physicochemical properties, nanoparticles (NPs) with various compositions (metals, metal oxides, semiconductors, and organic molecules) are widely used in various materials, including those in the consumer product, architecture, energy, and biomedicine fields (De Volder et al., 2013; Peng et al., 2014; Lane et al., 2015; Chen et al., 2016). NPs may enter the human body through oral administration, implantation, intravenous injection, transdermal absorption, etc., eliciting public concerns about the adverse effects of NPs (Zhang et al., 2014). Once entering the human blood circulation system, NPs spontaneously adsorb proteins, which reduce their surface free energy (Cedervall et al., 2007; Lundqvist et al., 2008; Monopoli et al., 2012; Su et al., 2016). Moreover, NPs can interact with the cell membrane (Lin et al., 2010; Jing and Zhu, 2011; Lesniak et al., 2013), lipids (Leroueil et al., 2008), proteins (Mahmoudi et al., 2011), and DNA (Asharani et al., 2009; Xie J. et al., 2019), eliciting various bioeffects, such as the disruption of the cell membrane, oxidative stress, apoptosis, inflammation, and autophagy, which may ultimately lead to cytotoxicity. Elucidation of NP-induced cytotoxicity is crucial for human health.

Interactions between NPs and biosystems are critically determined by surface chemistry. Surface modification of NPs may be made during the synthesis process or environmental exposure (Bystrzejewska-Piotrowska et al., 2009; Wang et al., 2013). For example, charged ligands such as cetyl trimethyl ammonium bromide (CTAB) and citrate acid are frequently used as protective agents in NP synthesis processes (Nikoobakht and El-Sayed, 2003; Lim et al., 2009). Surface decorations can regulate the physicochemical properties of NPs to ultimately affect their various biological effects. For example, a decoration comprising positively charged and hydrophobic ligands on NPs can enhance the cellular uptake level (Su et al., 2012; Li et al., 2015a). A decoration consisting of poly(ethylene glycol) (PEG) can reduce protein binding in the blood and avoid recognition by the reticular endothelial system (RES) (Zhang T. et al., 2019). Moreover, surface chemistry is one of the key factors that can trigger adverse effects in biosystems. The role of surface functionality in determining nanobiological interactions has been well reviewed in some excellent recent literature (Verma and Stellacci, 2010; Albanese et al., 2012; Kim et al., 2013; Nam et al., 2013; Zhu et al., 2013). To establish a distinct relationship between the surface chemistry of NPs and cytotoxicity, in this review, we systematically summarize our research and that of others on the latest progress in understanding the impact of typical surface chemistry, including surface charge, hydrophobicity, and combinatorial decorations, on various cytotoxicity-related bioeffects, including protein binding, cellular uptake, oxidative stress, autophagy, inflammation, and apoptosis. The surface chemistry driver of bioeffect levels and molecular pathways is discussed in detail. These findings may be used to predict adverse cellular responses of NPs and guide the design of environmentally safe NPs, including medical NPs that could potentially be used in the treatment of diseases.

## Protein Binding

NPs can enter the human body through multiple pathways and be distributed to different organs through circulating blood. In blood, NPs progressively and selectively adsorb proteins and form protein coronas that reduce their surface free energy. According to binding strength and relative position, a protein corona can be divided into a hard part and a soft part. The hard corona binds to the surface of the NP tightly, where it forms a near-monolayer. The soft corona is formed over top of the hard corona, and the interactions between the soft corona and NPs are weak (Monopoli et al., 2012). The protein corona on NPs can be analyzed by atomic force microscope images, fluorescence spectroscopy, circular dichroism, sodium dodecyl sulfate-polyacrylamide gel electrophoresis (SDS-PAGE), and mass spectrometry (Cedervall et al., 2007; Ge et al., 2011).

The formation of protein corona can result in undesired cellular uptake, nanoparticle aggregation, or immune responses (Karmali and Simberg, 2011). However, it can also alleviate cytotoxicity. For example, in THP-1 and human umbilical vein endothelial cells (HUVECs), the adsorption of bovine fibrinogen, bovine serum albumin (BSA), transferrin, or gamma globulin formed compact layers on NPs' surface, which effectively shield cells from the exposure of NPs' surfaces and alleviate the cytotoxicity induced by single-walled carbon nanotubes (SWCNTs) (Ge et al., 2011). In another study, serum protein adsorption significantly reduced the cytotoxicity of graphene oxide (GO) in A549 cells (Hu et al., 2011). In addition, protein binding can drastically alter the physicochemical properties of NPs and affect their behavior *in vivo* (Albanese et al., 2012).

In this section, we summarize recently published literatures related to the regulation of protein binding by NPs' surface charge, hydrophobicity, and combinatorial surface modifications (Table 1), which are generally considered as important factors that characterize NPs' surface coating.

**Table 1:** Protein binding regulated by NPs' surface chemistry.

**Composition**	**Size**	**Surface chemistry/Zeta potential/LogP**	**Protein binding capacity**	**References**
Gold	5–20 nm	Poly(N-(2-aminoethyl) acrylamide) (PAEA, 46– 57 mV), poly(acrylic acid) (PAA, −25 to −60 mV), poly(N-(2,3-dihydroxypropyl)acrylamide) (PDHA, slightly negatively charged)	PAEA>PAA>PDHA	Deng et al., 2012
Gold	17 nm	Methoxy-PEG-alkyl-thiol, methoxy-PEG-thiol	Methoxy-PEG-thiol>methoxy-PEG-alkyl-thiol	Larson et al., 2012
Gold	14–22 nm	Mercaptosuccinic acid, N-4-thiobutyroil glucosamine, PEG_5000_ and alkyl-PEG_600_	(Mercaptosuccinic acid and N-4-thiobutyroil glucosamine)>(PEG_5000_ and alkyl-PEG_600_)	Silvestri et al., 2017
Gold	5 nm	PEG, citric-, phosphine-, poly(isobutylene-alt-maleic anhydride)	[Citric-, phosphine-, poly(isobutylene-alt-maleic anhydride)]>PEG	Johnston et al., 2017
Gold	13 nm	PEG (−13.5 mV), tannic acid (−28.1 mV)	Tannic acid>PEG	Braun et al., 2016
Gold	15, 30, 60, 90 nm	PEG (5 kDa)	Negatively correlated with PEG density	Walkey et al., 2012
Gold	45 nm	PEG (2, 5, 10, 20 kDa), −5.4 mV to −25.4 mV	Positively correlated with PEG chain length	Su et al., 2018
Silver	30 nm	PEG (−16.2 mV), citrate (−22.9 mV), polyvinylpyrrolidone (−22.1 mV)	(Citrate, polyvinylpyrrolidone)>PEG	Pang et al., 2016
Silica	50 nm	COOH (−42 mV), NH_2_ (25 mV)	COOH>NH_2_	Kurtz-Chalot et al., 2017
Silica	100 nm	Hydration (−34.63 mV), dextran (−17.54 mV), gelatin (−23.52 mV), amination (14.91 mV), PEI (15.84 mV),	(Hydration, dextran, gelatin)>(amination, PEI)	Wang et al., 2017
Silica	170 nm	Succinic anhydride (−5 mV to −40 mV)	Decreased with the increase in negative charge density	Beck et al., 2017
Iron oxide	10 nm	Methacrylic acid, citric acid	Methacrylic acid>citric acid	Mekseriwattana et al., 2019
Iron oxide	12 nm	Glucose, PEG	Glucose>PEG	Stepien et al., 2018
ZnO	39 nm	PEG (10 mV), bare (30 mV)	Bare>PEG	Luo et al., 2014
SWCNTs	Diameter: 6-8 nm	COOH (hydrophilic, −23.27 mV), CH_3_ (hydrophobic, 12.63 mV)	COOH>CH_3_	Li et al., 2013
MWCNTs	Diameter: 10–20 nm, length: 5–15 μm	Pristine (−14.97 mV), PEG (−15.60 mV)	Pristine>PEG	Zhang T. et al., 2019
Nanodiamonds	5 nm	Hydrogen-terminated (49 mV), oxygen-terminated (−51 mV)	Oxygen-terminated> hydrogen-terminated	Aramesh et al., 2015
Polystyene	140 nm	COOH (−7.21 mV), NH_2_ (7.58 mV)	COOH>NH_2_	Kokkinopoulou et al., 2017
Polystyrene	50 nm	Sulfonated (−13.3 mV), carboxylated (−10.2 mV)	Carboxylated>sulfonated	Abdelkhaliq et al., 2018
N-isopropylacrylamide-co-N-tert-butylacrylamide copolymer	70 nm	NIPAM:BAM=85:15 (more hydrophilic), 65:35, and 50:50 (more hydrophobic)	50:50>65:35>85:15	Cedervall et al., 2007

### Surface Charge

Compared to neutral NPs, charged NPs tend to adsorb more proteins from serum. For example, poly(N-(2-aminoethyl)acrylamide) and poly(acrylic acid)-decorated gold nanoparticles (GNPs, 5–20 nm), which exhibit positive and negative charge, respectively, adsorbed large amounts of plasma proteins; however, relatively few proteins adsorbed onto neutrally charged poly(N-(2,3-dihydroxypropyl)acrylamide)-GNPs (Deng et al., 2012).

Regarding charged NPs, positive and negative charges exhibit different protein binding capacities. For example, silica NPs (50 nm) decorated with amine or carboxyl groups exhibited different protein binding levels, with the negatively charged silica NPs adsorbing more proteins from human serum than were adsorbed by their positively charged counterparts (Kurtz-Chalot et al., 2017). In another study, it was also found that polystyrene (PS) NPs (140 nm) decorated with carboxyl or amine group exhibited different binding capacities to human serum protein. The total number of corona proteins on carboxyl-decorated PS NPs was higher than the number on their amine-decorated counterparts (Kokkinopoulou et al., 2017). The surface charge of silica NPs (100 nm) affected the recruitment of transforming growth factor (TGF)-β1 to the NP surface. Positively charged aminated- and polyetherimide (PEI)-silica NPs completely failed to adsorb TGF-β1 in a mouse lung tissue homogenate supernatant, while negatively charged hydrogenated, dextran-silica, and gelatin-silica NPs largely adsorbed TGF-β1 (Wang et al., 2017). The surface charge of nanodiamonds (5 nm) could regulate protein binding speed. The adsorption rate of BSA on negatively charged nanodiamonds is higher than the rate on positively charged counterparts (Aramesh et al., 2015). Therefore, negatively charged NPs are more liable to adsorb proteins compared to positively charged NPs.

For NPs with a negative charge, the protein binding could also be tuned by other factors, including charge densities and ligand species. For example, libraries of silica NPs (170 ± 20 nm) with continuously changing surface charge densities were synthesized by tuning the surface density of negatively charged succinic anhydride ligand. The overall amounts of serum protein, BSA, and apolipoprotein 1 adsorbed onto silica NPs decreased with the increase in negative charge density (Beck et al., 2017). Both sulfonated and carboxylated PS NPs (50 nm) have negatively charged surfaces. Although the composition of the protein corona isolated from these PS NPs was the same, some proteins, including A2M, AFP, APOA2, APOH, and HBB were significantly less adsorbed onto the sulfonated PS NPs (Abdelkhaliq et al., 2018). In another study, it was found that negatively charged poly(methacrylic acid)-decorated iron oxide NPs (10 nm) tended to adsorb more proteins from fetal bovine serum (FBS) than were adsorbed by citric acid-decorated iron oxide NPs (Mekseriwattana et al., 2019).

In addition to protein levels, surface charge could also define the protein species adsorbed onto NPs. For example, more total and complement protein species were adsorbed on positively charged silica NPs (50 nm) than were adsorbed on their negatively charged counterparts (Kurtz-Chalot et al., 2017). In another study, the protein binding of amine-functionalized and bare PS NPs (100 nm) was compared. The proteins adsorbed on the positively charged PS NPs were more hydrophobic than those adsorbed on the bare PS NPs (Kendall et al., 2015). For pristine, amine-modified and carboxyl-modified silica NPs (22.4 ± 2.2 nm), the surface charge affects the adsorption of the proteins species related to immune responses, transport, regulation of proteolysis, hyaluronan metabolic processes, and other functions (Mortensen et al., 2013). Citric acid, poly(acrylic acid), and oleic acid decorations all endow iron oxide NPs with a negative charge; however, the protein corona composition and structure were influenced by these three kinds of surface decoration (Jedlovszky-Hajdú et al., 2012).

### Hydrophobicity

Cedervall et al. (2007) used a series of copolymer NPs to investigate the impact of NP hydrophobicity on protein binding. They found that the number of protein molecules adsorbed onto NPs increased with NP hydrophobicity. However, in other papers, an opposite conclusion was drawn. For example, hydrophilic COOH- and hydrophobic CH_3_-SWCNTs exhibited different affinities for recombinant human bone morphogenetic protein-2 (rhBMP-2). The amount of rhBMP-2 adsorbed onto the COOH-SWCNTs was higher than the amount adsorbed onto the CH_3_-SWCNTs (Li et al., 2013). In another study, incorporating an alkyl linker between the PEG and thiol moieties enhanced the hydrophobicity of the ligands, which resulted in a decrease in protein binding onto GNPs (17 nm) (Larson et al., 2012).

Due to the presence of hydrophilic group, such as ether bond in the middle of the polymer chain and hydroxyl at the end, PEG modification on NPs could reduce surface hydrophobicity (Kleemann et al., 2005; Sheng et al., 2009; Xiong et al., 2012; Jiang et al., 2016). PEG decoration on GNPs and silver NPs leads to lower protein binding compared to other decorations. For example, compared to mercaptosuccinic acid and N-4-thiobutyroil glucosamine, PEG_5000_ and alkyl-PEG_600_ decorations on GNPs (14–22 nm) induced lower levels of protein adsorption from FBS (Silvestri et al., 2017). In another comparative study, PEG-GNPs (5 nm) were found to adsorb fewer proteins than were adsorbed by citric-, phosphine-, or poly(isobutylene-alt-maleic anhydride)-GNPs (Johnston et al., 2017). Moreover, PEG decoration on GNPs (13 nm) was also found to bind fewer proteins than were bound by the tannic acid decoration (Braun et al., 2016). For silver NPs (30 nm), PEG decoration led to lower adsorption of BSA than was adsorbed by citrate and polyvinylpyrrolidone decorations (Pang et al., 2016).

**Figure 1:**
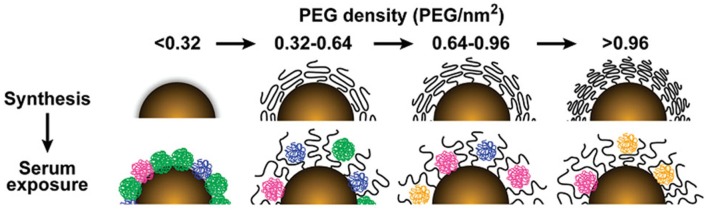
The influence of PEG density on serum protein adsorption to gold nanoparticles. The top panel shows as-synthesized gold nanoparticles grafted with PEG at increasing density. As PEG density increases, PEG volume decreases as a result of PEG–PEG steric interactions. The lower panel illustrates how PEG density determines the amount and relative abundance of serum proteins adsorbed to the gold nanoparticle surface after serum exposure. Adapted with permission from Walkey et al. (2012).

The PEG density and length can tune protein binding on GNPs. For GNPs of four different sizes (15, 30, 60, 90 nm), the total serum protein adsorption was negatively correlated with PEG density (Figure 1) (Walkey et al., 2012). Furthermore, as the molecular weight of the PEG decoration increased from 2 to 20 K, the amount of adsorbed protein on the GNPs (45 nm) showed an increasing trend (Su et al., 2018).

PEG decoration on other NPs can also reduce protein adsorption. For example, PEG decorated ZnO NPs (39 ± 4 nm) exhibited a lower level of protein adsorption from FBS compared to bare ZnO NPs (Luo et al., 2014). Compared to NPs with glucose decoration, PEG-iron oxide NPs (12 nm) bound fewer proteins (Stepien et al., 2018). PEG decoration was also found to reduce the adsorption of BSA and IgG compared to pristine multi-walled carbon nanotubes (MWCNTs, with an average diameter of 10–20 nm and length of 5–15 μm) (Zhang T. et al., 2019).

### Combinatorial Surface Modifications

Combinatorial chemistry could efficiently afford a vast number of structurally related molecules and materials. The synthesis of surface-modified nanoparticle libraries has been considered as a powerful tool to modulate nanoparticle properties (Zhou et al., 2008). Combining with both experimental and computational methods, the NP libraries could be used to rapidly discover nanoparticles with specific activity and reveal structure-activity relationship at the same time (Gao et al., 2011; Zhou et al., 2011; Wu et al., 2014; Liu Y. et al., 2015; Zhang et al., 2016).

For examples, Zhou et al. synthesized combinatorial libraries of surface-modified MWCNTs and GNPs to investigate the relationships between NP surface chemistry and their ability to bind proteins. They found that surface chemistry could tune the binding affinity of MWCNTs for four typical proteins, namely, BSA, carbonic anhydrase, chymotrypsin, and hemoglobin and for proteins in human plasma and a cell culture medium (Zhou et al., 2008). Furthermore, chemical modifications on the MWCNT surface could perturb the enzymatic activity of CYP3A4 in human liver microsomes by binding to the protein and altering its conformation. Based on a QSAR analysis, Zhang et al. found that long and complex hydrophobic or aromatic side chains on MWCNT surfaces were responsible for inducing the inhibitory effects of f-MWCNTs on CYP3A4, while pharmacophores with lower aromaticity and fewer tertiary nitrogen atoms were more likely to generate safe MWCNTs (Zhang et al., 2016). Moreover, Liu et al. also found that the surface chemistry of the GNPs could dramatically affect both non-specific binding (away from the peripheral site) and specific binding to acetylcholinesterase (AChE), which resulted in the inhibition of the enzyme (Liu Y. et al., 2015).

## Cellular Uptake

NPs can enter into cells through multiple pathways, including macropinocytosis and clathrin-, and caveolae-dependent endocytosis et al (Sahay et al., 2010). For macrophages, phagocytosis is the predominant mechanism, while non-professional phagocytes, including epithelial cells, fibroblasts, and endothelial cells, may also undertake phagocytosis relatively less frequently (Hillaireau and Couvreur, 2009). Multiple endocytosis inhibitors could be used to distinguish the uptake pathways, including cytochalasin D, methyl-β–cyclodextrin, nocodazole, etc. (Saha et al., 2013). However, non-specificity and cytotoxicity are inherent disadvantages of these inhibitors. Therefore, multiple inhibitors for a certain pathway are usually used simultaneously, and the dose is carefully chosen.

The cellular uptake level of NPs are usually determined by inductively coupled plasma-mass spectrometry and transmission electron microscopy (TEM) (Su et al., 2012; Saha et al., 2013; Wu et al., 2014; Van Haute et al., 2018). For fluorescent NPs and NPs decorated with fluorescent ligand, the uptake level and subcellular localization can also be analyzed by fluorescence microscope, laser scanning confocal microscopy, and flow cytometry (Zhang et al., 2002; Mahmoud et al., 2010).

The cellular uptake of NPs is strongly associated with various cytotoxicity-related bioeffects, including oxidative stress, apoptosis, autophagy, and inflammation (Xia et al., 2008; Foldbjerg et al., 2011; Stern et al., 2012; Sun et al., 2018). For example, Xia et al found that ZnO NPs (13 nm) internalized by RAW264.7 cells elicited oxidative stress, inflammation, and cell death (Xia et al., 2008). In our previous study, cellular uptake level of hydrophobic and positively charged GNPs (6 nm) was positively correlated with oxidative stress level in A549 cells. Inhibition of GNPs' internalization led to the decrease of oxidative stress level, indicating that oxidative stress induced by GNPs is internalization-dependent (Sun et al., 2018). Therefore, for certain NPs, cellular uptake is positively correlated with cytotoxicity. In this section, we summarize recently published literatures related to the regulation of cellular uptake by NPs' surface charge, hydrophobicity, and combinatorial surface modifications (Table 2).

**Table 2:** Cellular uptake regulated by NPs' surface chemistry.

**Composition**	**Size**	**Surface chemistry/Zeta potential/LogP**	**Cell line**	**Cellular uptake level**	**References**
Gold	18, 35, 65 nm	Ethanediamine, glucosamine, hydroxypropylamine, taurine, linear PEG	Primary human dermal microvascular endothelial cells	Ethanediamine>the rest decorations	Freese et al., 2012
Gold	33 × 30 nm, 55 × 14 nm	Poly(diallyldimethyl ammonium chloride) (50 mV), CTAB (40 mV), polystyrene sulfonate (−40 mV)	MCF-7	Poly(diallyldimethyl ammonium chloride)>CTAB>polystyrene sulfonate	Qiu et al., 2010
Gold	40, 80 nm	Polyethyleneimine (63.1 mV), lipoic acid (−73.3 mV)	HUVECs	Polyethyleneimine>lipoic acid	Chandran et al., 2017
Gold	10, 20, 40 nm	Cysteamine (26.33 to 47.12 mV), citrate (-29.28 to −38.4 mV), cysteine (0.13 to −0.92 mV)	Monocytes and macrophages	Cysteamine>(citrate, cysteine)	Oh and Park, 2014
Gold	15, 45 nm	Poly(allyamine hydrochloride) (20.9, 30.1 mV), PEG (−2.1, −1.0 mV)	SK-BR-3 breast cancer cells	Poly(allyamine hydrochloride)>PEG	Cho et al., 2010
Gold	20 nm	Poly(allylamine hydrochloride) (PAH, 16.6 mV), 1-palmitoyl-2-oleoyl-sn-glycero-3-phospho-L-serine/1-palmitoyl-2-hydroxy-sn-glycero-3-phosphocholine (L-PAH 48.7 mV, HL −51.9 mV)	Human dermal fibroblast cells	PAH>(L-PAH, HL)	Yang et al., 2014
Gold	6 nm	Lipoic acid and derivatives (−40 mV to 60 mV)	HeLa, HEK293, and A549	Positively correlated with positive charge density	Su et al., 2012; Sun et al., 2018
Gold	6 nm	Derivatives of lipoic acid (zeta potential: −5 mV to −20 mV, LogP: −2.7 to 2.4)	HEK293, A549, THP-1	Hydrophobic GNP>hydrophilic GNP	Li et al., 2015a; Sun et al., 2018
TiO_2_	50–65 × 8 nm	NH_2_ (35.2 mV), COOH (-20.9 mV)	Rat bone marrow mesenchymal stem cells	NH_2_ >COOH	Shrestha et al., 2016
TiO_2_	300 nm	PEG, pristine	A549, H1299	Pristine>PEG	Tedja et al., 2012
TiO_2_	length: 50–65 nm, width: 8 nm	PEG (−25.8 mV), NH_2_ (35.2 mV), COOH (−20.9 mV)	Rat bone marrow mesenchymal stem cells	(NH_2_, COOH)>PEG	Shrestha et al., 2016
ZnO	15 nm	1,2-dioleoyl-sn-glycero-3- phosphocholine) (DOPC), NH_2_	HeLa	DOPC>NH_2_	Dumontel et al., 2017
ZnO	10–30 nm	3- aminopropyltrimethoxysilane (APTES), pristine	HepG2	3- aminopropyltrimethoxysilane (APTES)>pristine	Bartczak et al., 2015
ZnO	39 nm	APTES (40 mV), pristine (30 mV)	THP-1 and differentiated THP-1 cells	APTES = pristine	Luo et al., 2014
ZnO	39 nm	PEG (10 mV), 3-aminopropyltriethoxysilane (APTES, 40 mV)	THP-1, differentiated THP-1	APTES>PEG	Luo et al., 2014
Silica	20, 30, 50, 80 nm	Amine, L-Ser, pristine	A549	Amine>(L-Ser, pristine)	Ojea-Jiménez et al., 2016
Silica	50 nm	NH_2_ (25 mV), COOH (−42 mV)	RAW264.7	NH_2_ = COOH	Kurtz-Chalot et al., 2017
Silica	50 nm	PEG (−29 mV), COOH (−42 mV), NH_2_ (25 mV)	RAW264.7	(COOH, NH_2_)>PEG	Kurtz-Chalot et al., 2017
Iron oxide	20 nm	PEG (−29.74 mV)	HUVECs, macrophages	Inhibited cellular uptake	Orlando et al., 2015
Iron oxide	150 nm	Carboxymethyl dextran (CMX, −11.6 mV), PEG (−10.6 mV)	Microglia, astrocytes, oligodendrocyte precursor cells, neural stem cells	CMX>PEG	Jenkins et al., 2016
SWCNTs	Length: 240 nm	NH_2_ (52.8 mV), COOH (−66.8 mV)	HeLa	NH_2_ >COOH	Budhathoki-Uprety et al., 2017
MWCNTs	Diameter: 10–20 nm, length: 5–15 μm	COOH (−31.93 mV), pristine (−14.97 mV)	RAW264.7	COOH>pristine	Zhang T. et al., 2019
MWCNTs	Diameter: 10–20 nm, length: 5–15 μm	PEG (−15.6 mV), pristine (−14.97 mV)	RAW264.7	Pristine>PEG	Zhang T. et al., 2019
Cellulose	10–20 × 120–300 nm	Rhodamine B isothiocyanate (RBITC, 8.7 mV), FITC (−46.4 mV)	HEK293	RBITC>FITC	Mahmoud et al., 2010
Polymer	<100 nm	Poly[2-(diisopropylamino)ethyl methacrylate], PEO	Telo-RF	Poly[2-(diisopropylamino)ethyl methacrylate]>PEO	De Castro et al., 2018
PLGA	170 nm	PEI (40 mV), BSA (−20 mV)	Human endothelial cells (CRL-1730)	PEI>BSA	Yu et al., 2012
Polystyrene	100 nm	NH_2_ (56 mV), COOH (−46 mV)	THP-1	NH_2_ = COOH	Lunov et al., 2011
QDs	6 nm	Lipoic acid (−15 mV) and derivatives (zwitterionic −5 mV, cationic 20 mV)	HeLa	(Cationic, anionic)>zwitterionic	Park et al., 2011

### Surface Charge

**Figure 2:**
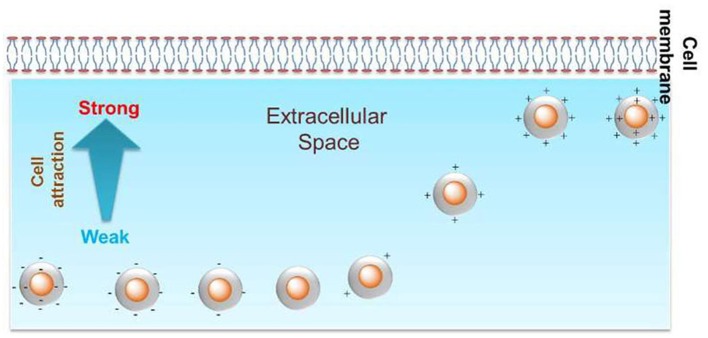
Positive charge density could tune the cellular uptake level of GNPs in HeLa cells. Reproduced with permission from Su et al. (2012).

The surface charge of GNPs can tune the cellular uptake level. In primary human dermal microvascular endothelial cells, positively charged ethanediamine-decorated GNPs (18, 35, and 65 nm) were internalized to a greater extent than were neutral or negatively charged GNPs (Freese et al., 2012). In another study, the zeta potential of poly(diallyldimethyl ammonium chloride) (PDDAC), CTAB, and polystyrene sulfonate (PSS)-decorated gold nanorods (GNRs) (33 × 30 and 55 × 14 nm) in aqueous solutions was found to decrease from ~50 to −40 mV, and the cellular uptake level by these GNRs in MCF-7 cells decreased with the decrease in zeta potential (Qiu et al., 2010). Positively charged branched polyethyleneimine-decorated GNPs (40, 80 nm) were more likely than negatively charged lipoic acid-GNPs to be endocytosed by HUVECs (Chandran et al., 2017). In monocytes and macrophages, positively charged cysteamine-GNPs (10, 20, and 40 nm) were internalized at a higher level than were negatively charged or zwitterionic GNPs (Oh and Park, 2014). More GNPs (15 and 45 nm) decorated with positively charged poly(allyamine hydrochloride) than neutral GNPs were internalized in SK-BR-3 breast cancer cells (Cho et al., 2010). More cationic poly(allylamine hydrochloride)-coated GNPs (20 nm) than anionic GNPs underwent endocytosis in human dermal fibroblast cells (Yang et al., 2014). A library of GNPs (6 nm) with continuously changing positive charge/negative charge density was constructed by varying the ratio of positively/negatively charged ligands to neutrally charged ligands. In HeLa, HEK293, and A549 cells, Su et al. (2012)and Sun et al. (2018) found that the level of GNP endocytosis was positively correlated with a positive charge density, while negative charge density had no significant influence on cellular uptake (Figure 2). Therefore, in multiple immune and non-immune cells, positively charged GNPs are more prone to endocytosis than are neutral and negatively charged GNPs.

**Figure 3:**
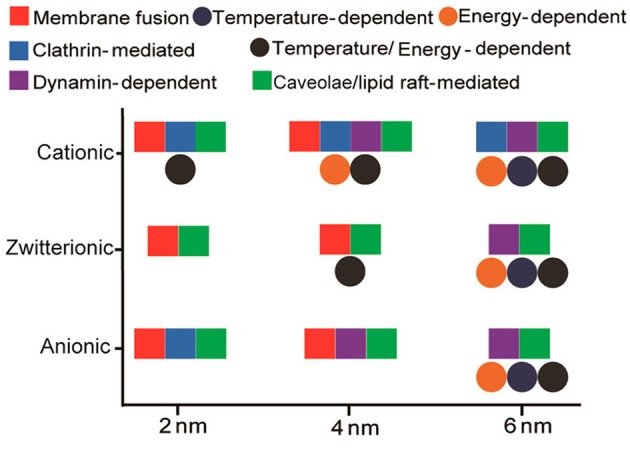
Interplay of size and surface functionality on the cellular uptake pathway of GNPs. Reproduced with permission from Jiang et al. (2015).

In addition to the cellular uptake level, the surface charge can also tune the endocytosis pathway of GNPs. For example, in HeLa cells, positively charged GNPs (2, 4, and 6 nm) were endocytosed through multiple pathways, including the clathrin- and caveolae/lipid raft-dependent pathways. Zwitterionic GNPs (2 and 4 nm) were prone to entering cells through membrane fusion, and zwitterionic GNPs (6 nm) were endocytosed through the caveolae/lipid raft-mediated pathway. Negatively charged GNPs (2 and 4 nm) displayed a similar endocytosis pathway to that of positively charged GNPs, while negatively charged GNPs (6 nm) were internalized through the caveolae/lipid raft-mediated pathway (Figure 3) (Jiang et al., 2015). GNRs (aspect ratio = 3) exhibited different endocytosis mechanisms in HaCaT cells on the basis of their decoration with positively or negatively charged ligands. Positively charged peptide-GNRs were internalized mainly through macropinocytosis and clathrin-mediated endocytosis, while negatively charged COOH-GNRs were endocytosed through macropinocytosis and caveolae-related mechanisms (Untener et al., 2013).

The surface charge of oxide NPs can impact the level of their cellular uptake. For example, positively charged NH_2_-TiO_2_ NRs (50–65 × 8 nm) were endocytosed by rat bone marrow mesenchymal stem cells at a much higher level than were negatively charged COOH-TiO_2_ NRs (Shrestha et al., 2016). ZnO NPs (15 nm) decorated with both (1,2-dioleoyl-*sn*-glycero-3-phosphocholine) (DOPC) and amine-propyl chains exhibited a positive charge in water. However, DOPC-ZnO NPs were internalized at a significantly higher level than were NH_2_-ZnO NPs in HeLa cells, a distinction that may be due to the difference in positive charge (Dumontel et al., 2017). Moreover, the uptake level that is affected by surface charge may vary with cell type. For example, positively charged silica NPs (20, 30, 50, 80 nm) are more likely to be endocytosed than their pristine or negatively charged counterparts in A549 cells (Ojea-Jiménez et al., 2016); however, in RAW264.7 cells, 50 nm silica NPs with either a positive or a negative charge exhibited similar uptake levels (Kurtz-Chalot et al., 2017). In HepG2 cells, the cellular uptake of positively charged 3-aminopropyltrimethoxysilane (APTES)-ZnO NPs (10–30 nm) was greater than the uptake of pristine ZnO NPs (Bartczak et al., 2015). However, in THP-1 and differentiated THP-1 cells, the cellular uptake of positively charged APTES-ZnO NPs (39 nm) was similar to that of pristine ZnO NPs (Luo et al., 2014).

In addition to the uptake level, the surface charge of oxide NPs can also modulate the uptake pathway. For example, an enhanced carboxyl group ratio on iron oxide NPs (33–45 nm) led to an increase in the negative charge density. In CaCo-2 cells, iron oxide NPs with a lower negative charge density were prone to internalization through a macropinocytosis mechanism, while iron oxide NPs with a higher negative charge density tended to be endocytosed through clathrin- and caveolae-dependent pathways (Ayala et al., 2013).

The surface charge can tune the cellular uptake level and pathway of carbon-based NPs. Positively charged amine-SWCNTs (mean length of 240 nm) were internalized at a higher level than were negatively charged carboxy-SWCNTs (mean length of 177 nm) in HeLa cells cultured in complete media. The absence of serum in the medium did not affect the uptake of positively charged amine-SWCNTs; however, the absence of serum significantly enhanced the internalization of negatively charged carboxy-SWCNTs in HeLa cells (Budhathoki-Uprety et al., 2017). In RAW264.7 cells, the cellular uptake of carboxyl-MWCNTs (average diameter of 10–20 nm and length of 5–15 μm) was greater than that of pristine MWCNTs, and the BSA and IgG corona could alleviate the internalization of the carboxy-MWCNTs and enhance the uptake of the pristine MWCNTs (Zhang T. et al., 2019). The internalization of carbon NPs (CNPs) smaller than 50 nm was systematically investigated in breast cancer cells in different stages (including the early and late metastatic stages). The internalization levels of anionic and neutral CNPs were higher than the level of cationic CNPs as the cancer progressed from the early to late metastatic stage, and the endocytosis pathway was also different for the positively/negatively/neutrally charged CNPs in different cancer stages (Srivastava et al., 2017).

For polymer NPs, the surface charge can also affect cellular uptake. Cellulose nanocrystal (CNC) is a novel material applied in regenerative medicine and drug delivery. CNCs (10–20 × 120–300 nm) with a positive surface charge were significantly endocytosed by HEK293 cells, while negatively charged CNCs were not significantly internalized by these cells (Mahmoud et al., 2010). NPs decorated with zwitterionic poly[2-(diisopropylamino)ethyl methacrylate] ligands were internalized at a higher level than their neutral counterparts (De Castro et al., 2018). Poly(D,L-lactide-coglycolide) (PLGA) is widely used in biomedical fields. The level to which human endothelial cells (CRL-1730) internalized PLGA NPs (170 nm) coated with positively charged PEI ligand was higher than the level to which they internalized BSA-PLGA NPs. The endocytosis mechanism was similar for both kinds of surface decorations, with macropinocytosis and clathrin-mediated endocytosis being the predominate mechanisms (Yu et al., 2012). The uptake level of NH_2_-PS NPs (100 nm) and COOH-PS NPs (100 nm) by THP-1 cells were similar, while COOH-PS NPs were more likely to be endocytosed by macrophages than were NH_2_-PS NPs. Moreover, the internalization mechanism was different for the NH_2_-PS and COOH-PS NPs taken up by macrophages compared to mechanism by which they were internalized by THP-1 cells, indicating that cell type should also be considered in determining cellular uptake (Lunov et al., 2011).

The surface charges of quantum dots (QDs) can determine the cellular uptake levels and pathways. Positively and negatively charged QDs could be massively endocytosed by HeLa cells, while QDs with zwitterionic surfaces were internalization to a lesser extent. Moreover, positively charged QDs were endocytosed by energy-dependent and energy-independent pathways, while negatively charged QDs were only endocytosed by energy-dependent pathways (Park et al., 2011). Cationic CdSe/ZnS QDs (4–5 nm) induced clathrin-mediated endocytosis in HeLa cells, while zwitterionic–lipophilic QDs mainly interacted with lipid rafts in the cell membrane, which led to lipid raft-mediated endocytosis (Chakraborty and Jana, 2015). In another study, COOH-PEG-QDs were found to be internalized through lipid raft- and SRA-mediated mechanisms in A549 cells, which were associated with the activated NF-κB pathway, while NH_2_-PEG-QDs were mainly internalized through lipid raft-mediated endocytosis and activated p38 MAPK/AP-1 signaling cascades (Zhang et al., 2013).

### Hydrophobicity

**Figure 4:**
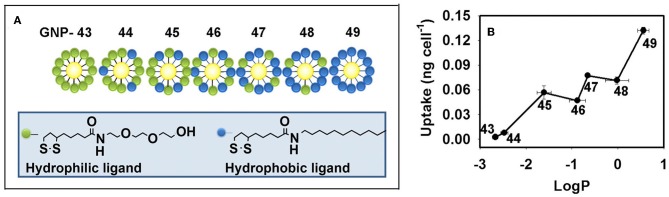
**(A)** GNP library with a continuous change in hydrophobicity. **(B)** Hydrophobicity regulates the cellular uptake level of GNPs in macrophages. Adapted with permission from Li et al. (2015a).

The hydrophobicity of GNPs can tune the cellular uptake level. By varying the ratio of hydrophobic ligands to hydrophilic ligands on the NP surface, a GNP library (6 nm) with continuously changing hydrophobicity was synthesized. In HEK293, A549, and THP-1 cells, the cellular uptake levels were found to be positively correlated with the hydrophobicity of the GNPs (Figure 4) (Li et al., 2015a; Sun et al., 2018).

To improve the bioavailability of NPs, PEG has been frequently used to modify the NP surfaces. PEG decorations could enhance NP hydrophilicity, dispersibility, and inhibit opsonization, thus significantly reducing the internalization of various types of NPs. For example, PEG reduced the uptake level of iron oxide NPs (~20 nm) in HUVECs and macrophages, and this PEG decoration-reduced uptake was more noticeable in macrophages than it was in HUVECs (Orlando et al., 2015). In another study, PEG decoration on iron oxide NPs alleviated the internalization in multiple (immune and non-immune) brain cell types, including microglia, astrocytes, oligodendrocyte precursor cells, and neural stem cells (Jenkins et al., 2016). PEG decoration on positively and negatively charged silica NPs (50 nm) reduced the level of NP internalization into RAW264.7 cells (Kurtz-Chalot et al., 2017). Few TiO_2_ NPs decorated with PEG-like polymers were internalized into A549 and H1299 cells (Tedja et al., 2012). In another study, PEG decorated TiO_2_ NRs (50–65 nm in length and 8 nm in width) were internalized to a lesser extent into rat bone marrow mesenchymal stem cells compared to the internalized levels of NRs with NH_2_ or COOH decorations (Shrestha et al., 2016). PEG decoration on ZnO NPs (39 nm) reduced the internalization into THP-1 and differentiated THP-1 cells compared to levels of NPs with APTES decoration that were internalized (Luo et al., 2014). Regarding MWCNTs (average diameter of 10–20 nm and length of 5–15 μm), PEG decoration reduced the number taken up by RAW264.7 cells (Zhang T. et al., 2019).

### Combinatorial Surface Modifications

The impact of surface chemistry on cellular uptake was investigated based on a combinatorial MWCNT library. Gao et al. found that modification of COOH-MWCNTs could redirect them from binding to mannose receptors to binding to scavenger receptors (Gao et al., 2011). In addition, the impact of surface chemistry on folate targeting was investigated by Zhou et al., who used a dual-ligand GNP array to assess Hela, KB, and HepG2 cells. The secondary ligand on GNPs may interact with the receptors surrounding the folate receptors according to their different intensities, resulting in a specific, unique level of cellular uptake (Zhou et al., 2011).

## Oxidative Stress

Reactive oxygen species (ROS), including ${\text{O}}_{2}^{-}$, OH^.^, and H_2_O_2_, are the derivatives of oxygen in physiological environments. ROS are generated mainly from mitochondria and NADPH oxidase in cells. The electron leakage in the mitochondrial respiratory chain is captured by oxygen, which leads to ROS generation (Murphy, 2009). NADPH oxidase is found in both phagocytic and non-phagocytic cells. Parts of the subunits are located on the cell membrane, and the other parts are located in the cytoplasm under quiescent conditions. Once activated, subunits in the cytoplasm will translocate to the cell membrane, resulting in the assembly of all the subunits (Bedard and Krause, 2007). The overproduction of ROS leads to oxidative stress, which is considered to be a main mechanism of nanotoxicity in recent years (Nel et al., 2006).

Intracellular ROS and oxidative stress could be determined by various methods. For example, fluorescent probes, including dichlorodihydrofluorescein and its derivatives, dihydroethidium, MitoSOX™ Red, etc., are the most convenient and widely used methods (Xia et al., 2006; Karlsson et al., 2008; Passagne et al., 2012). According to the hierarchical oxidative stress model, ROS stimulate the production of antioxidases. Therefore, the expression of an antioxidase, such as heme oxygenase-1, could be used to detect oxidative stress (Nel et al., 2006; Yu et al., 2012). Moreover, intracellular ROS induce a decrease in the GSH level and GSH/GSSG ratio, which are also used as oxidative stress markers (Piao et al., 2011; Nguyen et al., 2013). In this section, we summarize recently published literatures related to the regulation of oxidative stress by NPs' surface chemistry (Table 3).

**Table 3:** Oxidative stress regulated by NPs' surface chemistry.

**Composition**	**Size**	**Surface chemistry/Zeta potential/LogP**	**Cell line**	**Oxidative stress level**	**References**
Gold	6 nm	Lipoic acid and derivatives (−40 mV to 60 mV)	HEK293, A549	Positively correlated with positive charge density	Sun et al., 2018
Gold	55.7 × 13.2 nm	PEI, poly sodium-p-styrene sulfonate (PSS)	A549	PEI>PSS	Liu et al., 2016
Gold	6 nm	Derivatives of lipoic acid (zeta potential: −5 mV to −20 mV, LogP: −2.7 to 2.4)	HEK293, A549	Hydrophobic GNP>hydrophilic GNP	Sun et al., 2018
Gold	2 nm	Hydrophobic alkyl ends	HeLa	Positively correlated with the length of alkyl	Chompoosor et al., 2010
ZnO	29 nm	Pristine (4.49 mV), oleic acid (10.15 mV), poly(methacrylic acid) (-40.21 mV)	WIL2-NS human 364 lymphoblastoid cells	Pristine>[oleic acid, poly(methacrylic acid)]	Yin et al., 2010
ZnO	98 nm	3-Aminopropyl triethoxysilane (APTES, 11.1 mV), pristine (-30.4 mV)	A549, human skin fibroblasts (HSFs)	APTES>pristine	Keleştemur et al., 2017
Iron oxide	70 nm	Glucose, citric acid	CT26 colorectal cancer cells	Glucose>citric acid	Wydra et al., 2015
TiO_2_	60 × 80 nm	Pristine (21.4 mV), NH_2_ (35.2 mV), COOH (-20.9 mV)	Rat bone marrow mesenchymal stem cells	(Pristine, NH_2_)>COOH	Shrestha et al., 2016
Silica	45 nm	3-trihydroxysilyl)propylmethyl-phosphonate (THPMP, −52 mV), N-Trimethoxysilylpropyl-N,N,N-trimethylammonium chloride (TMAC, 38.9 mV)	RAW264.7	THPMP>TMAC	Liu T.-P. et al., 2015
Silica	50 nm	Hydrophobic linker/ hydrophilic linker (-1.53 mV to −13.6 mV)	RAW264.7	Hydrophobic linker>hydrophilic linker	Chen et al., 2017
MWCNTs	N.A.	COOH, neutral ligand	Macrophage	COOH>neutral ligand	Gao et al., 2011
MWCNTs	Diameter: 20–30 nm	COOH, pristine	HUVECs	COOH = pristine	Long et al., 2018
Graphene	<50 nm	Graphene oxide (−8.3 mV), carboxyl grapheme (−55.1 mV)	HepG2	Graphene oxide = carboxyl grapheme	Lammel et al., 2013
Graphene	200 nm	PEI (40.4 mV), pristine (−7.36 mV)	macrophage	PEI>pristine	Luo et al., 2015
Nanodiamonds	5 nm	OH (−12.2 mV), pristine (1 mV)	A549	OH = pristine	Solarska-Sciuk et al., 2014
Nanodiamonds	6–7 nm	NH_3_^+^ (0.3 mV), COOH (−37.3 mV)	Rat bone mesenchymal stem cells	NH_3_^+^ >COOH	Zhang Y. et al., 2019
Nanodiamonds	50–60 nm	COOH (−25 mV), PVP (−15 mV), OH (−10 mV), imidazolium (IM, 10 mV), tertiary methyl ammonium ethyl methacrylate cation (TMAEA, 20 mV)	HeLa	IM>TMAEA>COOH, PVP, OH)	Vankayala et al., 2014
Polystyrene	50 nm	NH_2_ (43 mV), COOH (−46.7 mV), pristine (−50.5 mV)	Human alveolar epithelial type I-like cells (TT1), primary human alveolar macrophages, primary human alveolar type 2 (AT2) cells	NH_2_ >(COOH, pristine) for the first two cell lines. NH_2_ = COOH = pristine for the last cell line	Ruenraroengsak and Tetley, 2015
Polystyrene	60 nm	NH_2_ (40.3 mV), COOH (−27.6 mV)	RAW264.7	NH_2_ >COOH	Xia et al., 2006

*N.A., not available*.

### Surface Charge

**Figure 5:**
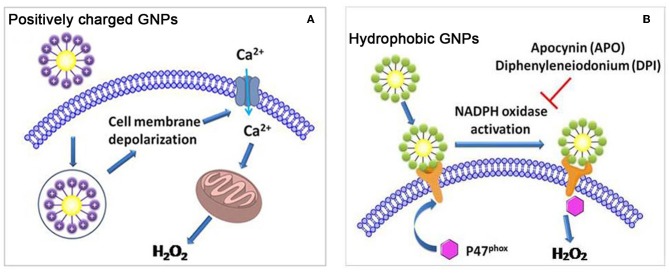
**(A)** Positively charged GNPs induce cell membrane depolarization and calcium channel opening, and stimulate mitochondria to generate intracellular oxidative stress. **(B)** Hydrophobic GNPs induce oxidative stress by perturbing NADPH oxidase. Adapted with permission from Sun et al. (2018).

To investigate the impact of surface charge on cellular oxidative stress, we constructed two libraries of GNPs (6 nm) that exhibit continuously changing in positive and negative charges. After exposing GNPs to A549 and HEK293 cells, we found that positive charge density was positively correlated with ROS level, while negative charge density exhibited no impact on intracellular ROS level. Studies on ROS-related mechanisms indicated that the cellular uptake of positively charged GNPs induced cell membrane depolarization and calcium channel opening and ultimately stimulated mitochondria to generate intracellular oxidative stress (Figure 5A) (Sun et al., 2018). In another study, it was also found that positively charged PEI-GNRs (55.7 × 13.2 nm) led to a decrease in intracellular GSH levels and GSH/GSSG ratios in A549 cells, while negatively charged PSS-GNRs exhibited no influence on GSH levels (Liu et al., 2016).

The surface charge of oxide NPs could also regulate the cellular oxidative stress level. For example, positively charged ZnO NPs (29 nm) induced the highest ROS level in WIL2-NS human lymphoblastoid cells, followed by negatively charged oleic acid and poly(methacrylic acid) decorated ZnO NPs (Yin et al., 2010). In A549 cells and human skin fibroblasts (HSFs), it was also determined that positively charged ZnO NPs (98 nm) were more prone to inducing ROS and mitochondria membrane depolarization than were their negatively charged counterparts (Keleştemur et al., 2017). Iron oxide NPs decorated with neutrally charged glucose ligands elicited higher ROS levels in CT26 colorectal cancer cells than did their citrate-decorated counterparts with a negative surface charge (Wydra et al., 2015). In rat bone marrow mesenchymal stem cells, pristine and NH_2_-TiO_2_ NRs (60 × 8 nm) induced ROS production, while COOH-TiO_2_ NRs had no significant impact (Shrestha et al., 2016). Therefore, it seems that the positively charged and neutral oxide NPs described above are more likely to induce ROS than are their negatively charged counterparts. However, in another study, it was found that negatively charged silica NPs induced ROS in RAW264.7 cells, while positively charged silica NPs had no influence on ROS production (Liu T.-P. et al., 2015).

COOH-MWCNTs induced higher ROS levels than neutrally charged MWCNTs induced in macrophages (Gao et al., 2011). However, in HUVECs, both pristine and COOH-MWCNTs elicited similar levels of ROS and decreased GSH levels (Long et al., 2018). As to graphene, GO and COOH-graphene induced similar amounts of ROS in HepG2 cells (Lammel et al., 2013). In macrophages, a positively charged PEI decoration on graphene led to the highest level of ROS, followed by a neutral decoration (Luo et al., 2015). As to nanodiamonds, the impact of surface charge on intracellular ROS level was also investigated. For example, decoration of negatively charged ligands on nanodiamonds (10 nm) could not enhance the ROS level in A549 cells (Solarska-Sciuk et al., 2014). However, in another paper, it was found that both positively and negatively charged nanodiamonds (6–7 nm) could elicit ROS production in rat bone mesenchymal stem cells, and the former induced higher ROS level than the latter (Zhang Y. et al., 2019). Moreover, Vankayala et al. also found that positively charged nanodiamonds (50–60 nm) induced higher ROS level than negatively charged, zwitterionic, and neutral nanodiamonds in HeLa cells (Vankayala et al., 2014).

Unmodified PS NPs and PS NPs with amine and carboxyl groups (50 nm) could regulate ROS levels in different cell lines. In human alveolar epithelial type I-like cells (TT1) and primary human alveolar macrophages, amine-PS NPs induced the highest ROS level, followed by unmodified and carboxyl-PS NPs. In primary human alveolar type 2 (AT2) cells, however, these three kinds of decorations elicited similar levels of intracellular ROS (Ruenraroengsak and Tetley, 2015). In another study, it was found that amine-PS NPs (60 nm) elicited mitochondrial ROS production, while carboxyl-PS NPs with the same size had no impact on intracellular ROS levels (Xia et al., 2006).

### Hydrophobicity

By varying the ratio of hydrophobic/hydrophilic ligands on the GNP surface, we synthesized a GNP library in which the NPs had continuously changing hydrophobicity. In A549 and HEK293 cells, GNP hydrophobicity was positively correlated with ROS level. Moreover, hydrophobic GNPs were found to elicit the translocation of P47^phox^ subunits to the cell membrane, leading to NADPH oxidase-dependent oxidative stress (Figure 5B) (Sun et al., 2018). In other studies, hydrophobic decorations on NPs were also more likely to elicit ROS production. For example, the length of hydrophobic alkyl ends on positively charged ligands was positively correlated with the ROS level induced by the GNPs (2 nm) in HeLa cells (Chompoosor et al., 2010). A hydrophobic linker decorated on silica NPs (50 nm) induced higher ROS production than did a hydrophilic linker decoration (Chen et al., 2017).

## Autophagy

Autophagy is a self-eating process that leads to the degradation of dysfunctional cellular components. Autophagy can be divided into three categories: macroautophagy, microautophagy, and chaperone-mediated autophagy (Levine and Kroemer, 2008; Mizushima et al., 2008). Various approaches can be used to determine autophagy the cell undergoes. For example, TEM can reveal the morphology of autophagic structures (Yu et al., 2014). The conversion of LC3-I to LC3-II is a biomarker of autophagy and is usually determined by Western blotting. Moreover, green fluorescent protein (GFP)-LC3 transfected cell lines can be used for high-throughput screening of autophagy (Wu et al., 2014). A high level of autophagy induced by NPs may lead to autophagy-related cell death or cytotoxicity (Chen et al., 2005; Liu et al., 2011). In this section, we summarize recently published literatures related to the regulation of autophagy by NPs' surface charge, hydrophobicity, and combinatorial surface modifications (Table 4).

**Table 4:** Autophagy regulated by NPs' surface chemistry.

**Composition**	**Size**	**Surface chemistry/Zeta potential/LogP**	**Cell line**	**Autophagy level**	**References**
Gold	N.A.	CTAB (40 mV), polystyrene sulfonate (PSS, −60 mV)	HCT116	CTAB>PSS	Wan et al., 2015
Gold	55 × 14 nm	CTAB, PSS, poly (diallyldimethylammonium chloride) (PDDAC)	A549, MRC-5	CTAB>(PSS, PDDAC)	Li et al., 2015b
Gold	10 nm	Hexane	HUVECs	Enhanced autophagy level	Manshian et al., 2014
ZnO	100, 130 nm	Triethoxycaprylylsilane (hydrophobic, −16.67 mV), pristine (−19.53 mV)	A549-macrophage co-culture	Triethoxycaprylylsilane<pristine	Liu et al., 2019
Graphene	3.5–5 nm	NH_2_, COOH	A549	NH_2_ >COOH	Xie Y. et al., 2019
Graphene	N.A.	Dodecylamine, sodiumdodecyl sulfate	RAW264.7	Dodecylamine>sodiumdodecyl sulfate	Park et al., 2015
SWCNTs	N.A.	COOH, PEG	A549	COOH>PEG	Liu et al., 2011

*N.A., not available*.

### Surface Charge

**Figure 6:**
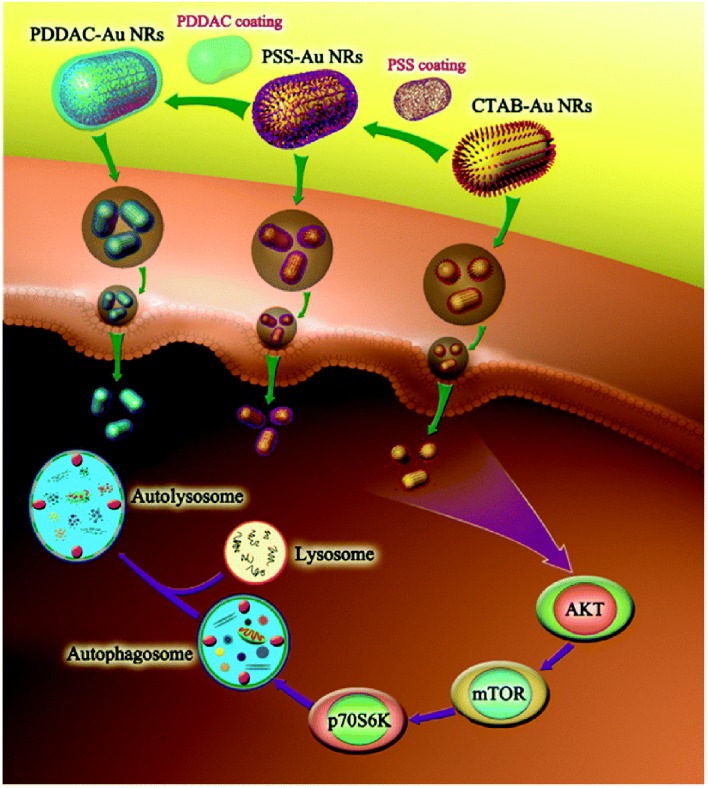
CTAB-GNRs induce autophagy through mTOR-dependent pathway while PSS- and PDDAC-GNRs do not cause an obvious autophagy process. Reproduced with permission from Li et al. (2015b).

Positively charged CTAB-GNRs could induce ROS and the transformation of LC3-I to LC3-II in HCT116 cells. However, negatively charged PSS decoration did not significantly induce autophagy (Wan et al., 2015). In another study, it was also found that CTAB-GNRs (55 × 14 nm) could induce autophagy, as evidenced by LC3-II conversion and p62 degradation, in A549 and MRC-5 cells. Moreover, the autophagy pathway stimulated by CTAB-GNRs is AKT-mTOR dependent. However, PSS and PDDAC decorations negligibly induced autophagy (Figure 6) (Li et al., 2015b). In addition to GNRs, the surface charge of carbon-based NPs can also tune autophagy levels and stimulate the related pathways. In A549 cells, NH_2_-graphene quantum dots (GQDs) induced cellular autophagy as evidenced by LC3 fluorescence tracking, LC3-II conversion, and autophagosome accumulation, while COOH-GQDs had no impact of autophagy (Xie Y. et al., 2019). In another study, the autophagy level induced by GO-decorated NPs with neutrally charged ligands was slightly higher than that of the negatively charged decorated NPs in RAW264.7 cells (Park et al., 2015). Therefore, it seems that positively and neutrally charged NPs are more likely to induce autophagy than are negatively charged NPs.

### Hydrophobicity

Hexane decoration on positively charged GNPs (10 nm) could elicit autophagy in HUVECs, and the number of hydrophobic hexanes in the ligand was positively correlated with the autophagy level (Manshian et al., 2014). However, hydrophobic ZnO NPs (130 nm) elicited lower expression of ATG7 gene than pristine ZnO NPs (100 nm) in A549-macrophage co-culture (Liu et al., 2019). COOH decorated SWCNTs induce autophagy-related cell death in A549 cells, while PEG decoration on SWCNTs could significantly reduce the autophagy level (Liu et al., 2011).

### Combinatorial Surface Modifications

We found that the surface modification on MWCNTs could tune autophagy in U87 and HEK293 cells. A total of 84 kinds of surface modification on MWCNTs could tune cell autophagy to various levels. Moreover, MWCNTs with diverse decorations could bind to different cell surface receptors and subsequently induced autophagy by activating diverse intracellular signaling pathways, including mTOR-dependent or -independent pathways (Wu et al., 2014).

## Inflammation

NP-induced bioeffects, including the induction of intracellular oxidative stress and the activation of receptors on cell membranes, may subsequently lead to the activation of the MAPK and NF-κB pathways, which results in the release of inflammation-related cytokines. A high level of inflammation could elicit cytotoxicity (Xia et al., 2006; Gao et al., 2011). In this section, we summarize recently published literatures related to the regulation of inflammation by NPs' surface charge, hydrophobicity, and combinatorial surface modifications (Table 5).

**Table 5:** Inflammation regulated by NPs' surface chemistry.

**Composition**	**Size**	**Surface chemistry/Zeta potential/LogP**	**Cell line/Animal model**	**Inflammation level**	**References**
Gold	50 × 15 nm	PEG-NH_2_, PEG-OH (−4.5 mV), PEG-COOH (−23.2 mV)	Primary human blood phagocytes	(PEG-OH, PEG-COOH)>PEG-NH_2_	Bartneck et al., 2010
Gold	10.4 nm	Methoxypoly-ethylene glycol-graf t-poly(L-lysine) copolymer (MPEG-gPLL, 3.7 mV)	Mice	Induced inflammatory lesions	Bogdanov et al., 2015
Gold	2 nm	Hyamine (LogP of end group: 0.63–5.35)	Splenocytes	Positively correlated with hydrophobicity	Moyano et al., 2012
Gold	35 × 10 nm	Mercaptohexadecanoic acid (−46.2 mV), PEG (−11.1 mV)	HaCaT	Mercaptohexadecanoic acid > PEG	Grabinski et al., 2011
Silica	50 nm	NH_2_ (25 mV), COOH (−42 mV), PEG (−29 mV)	RAW264.7	COOH>(NH_2_, PEG)	Kurtz-Chalot et al., 2017
TiO_2_ nanowires	Diameter: 200–400 nm	−1.6 to −15.9 mV	Mice	Positively correlated with zeta potential	Park et al., 2013
ZnO	100, 130 nm	Triethoxycaprylylsilane (hydrophobic, −16.67 mV), pristine (−19.53 mV)	A549-macrophage co-culture	Triethoxycaprylylsilane<pristine	Liu et al., 2019
Iron oxide	10 nm	Pristine, PEG	A549	Pristine>PEG	Griffete et al., 2012
Carbon	Diameter: 60–200	N.A.	IC-21 murine macrophages	Hydrophobic>hydrophilic	Chun et al., 2011
MWCNTs	Diameter: 20–30 nm	COOH, pristine	HUVECs	Pristine>COOH	Long et al., 2018
MWCNTs	N.A.	COOH (−13.8 mV), pristine (−9.76 mV)	C57Bl/6 alveolar macrophage	Pristine>COOH	Hamilton et al., 2013
MWCNTs	<500 nm	Pristine (−6.8 mV), COOH (−12.2 to −32.2 mV)	Mice	Pristine>COOH	Jain et al., 2011
MWCNTs	Diameter: 10–20 nm, length: 5–15 μm	Pristine (−15.5 mV), PEG (−12.8 mV)	Mice	Pristine>PEG	Zhang et al., 2017
Graphene	Thickness: <5 nm, diameter: <2 μm	NH_2_ (15.5 mV), COOH (−35 mV)	Rats	NH_2_ >COOH	Lee et al., 2017
Graphene	Lateral dimension of ~100–200 nm	COOH, PEG	Mice	COOH>PEG	Sasidharan et al., 2015
Liposome	100–150 nm	Cationic, neutral, anionic ligand	Mice	Cationic>(neutral, anionic)	Wei et al., 2015
Lipid	<200 nm	Cationic, anionic ligand	Rats	Cationic>anionic	Gabal et al., 2014
Nanogels	50–60 nm	PEG (-2.46 mV), poly(sulfobetaine) (PSB, −2.01 mV), and poly(carboxybetaine) (PCB, −1.89 mV)	PBMCs	(PSB, PCB)>PEG	Li et al., 2018
Polymer	160 nm	Polyvinyl acetate (−3 mV)	Mice	Positively correlated with hydrophobicity	Dailey et al., 2015
Polymer	20–25 nm	PEG (1.7 mV, 8.8 mV, 15.4 mV)	Mice	Negatively correlated with PEG length	Ibricevic et al., 2013

*N.A., not available*.

### Surface Charge

GNRs (50 × 15 nm) decorated with PEG-NH_2_, PEG-OH, or PEG-COOH were exposed to primary human blood phagocytes, and the release of proinflammatory cytokines was measured. PEG-COOH and PEG-OH could enhance the production of IL-1β and CCL2, while PEG-NH_2_ did not affect the production of IL-1β or CCL2. PEG-COOH could enhance the production of IL-6, and the remaining decorations exerted no influence on IL-6 production (Bartneck et al., 2010). In RAW264.7 macrophages, negatively charged silica NPs (50 nm) induced the highest secretion of proinflammatory TNF-α, followed by neutral and positively charged silica NPs (Kurtz-Chalot et al., 2017). Therefore, for these NPs, negatively charged decorations are more prone to elicit the release of proinflammatory cytokines than are positively charged decorations.

Surface decoration on QDs (8–10 nm), namely, negatively charged COOH-PEG or COOH, positively charged NH_2_-PEG, and neutrally charged HO-PEG and CH_3_O-PEG, could regulate the mRNA levels of IL-1β, TNF-α, and CCL5 in A549- and THP-1-derived macrophages. In A549 cells, COOH-PEG and COOH decorations enhanced the mRNA levels of IL-1β and CCL5, and COOH-PEG, NH_2_-PEG, and COOH decorations enhanced the mRNA levels of TNF-α. In macrophages, COOH-PEG, NH_2_-PEG, HO-PEG, and COOH decorations enhanced the expression of IL-1β; COOH-PEG and COOH decorations enhanced the expression of TNF-α; and COOH-PEG enhanced the expression of CCL5 (Zhang et al., 2013).

A neutral decoration on MWCNTs is more likely to elicit the secretion of proinflammatory cytokines than is a negatively charged decoration. For example, in HUVECs, pristine MWCNTs did not affect the secretion of TNF-α, while COOH-MWCNTs slightly inhibited the release of TNF-α (Long et al., 2018). In another study, pristine MWCNTs elicited higher levels of IL-1β and IL-18 secretion in the C57Bl/6 alveolar macrophage model than did COOH-MWCNTs (Hamilton et al., 2013).

The protein corona could also affect the secretion of proinflammatory cytokines. For example, without BSA or IgG coronas, COOH-MWCNTs induced higher levels of IL-1β and TNF-α release than the pristine MWCNTs in RAW264.7 cells. With BSA coronas, pristine MWCNTs induced higher levels of IL-1β than did COOH-MWCNTs, and both kinds of MWCNTs induced similar levels of TNF-α. With IgG coronas, pristine MWCNTs induced higher levels of TNF-α than did COOH-MWCNTs, and both kinds of MWCNTs induced similar levels of IL-1β (Zhang T. et al., 2019).

Except for these *in vitro* investigations mentioned above, a series of *in vivo* experiments were also conducted to investigate the impact of surface charge on inflammation. For example, positively charged GNPs (10.4 ± 2.5 nm) administrated in mice led to inflammatory lesions (Bogdanov et al., 2015). Rats treated with positively charged graphene nanoplatelets (<5 nm thick, <2 μm in diameter) induced greater pulmonary inflammation than negatively charged counterparts (Lee et al., 2017). Mice treated with cationic liposomes (100–150 nm) triggered pulmonary inflammation, while neutral and anionic liposomes exhibited normal pulmonary histology (Wei et al., 2015). Cationic nanostructured lipid carriers (NLCs, <200 nm) led to the diffusion of inflammatory cells in the underlying lamina propria, however, rats treated with anionic NLCs exhibited normal structure of the lining epithelium (Gabal et al., 2014). TiO_2_ nanowires (diameter: 200–400 nm) with different negative charges could regulate inflammation *in vivo*. TiO_2_ nanowires with lowest negative charge induced the highest level of cytokines in the BAL fluid of mice (Park et al., 2013).

### Hydrophobicity

Hydrophobic NPs are prone to inducing the release of cytokines. For example, in IC-21 murine macrophages, hydrophobic carbon fibers induced higher levels of IL-6 and TNF-α secretion than did hydrophilic carbon fibers (Chun et al., 2011). Ligand hydrophobicity on GNPs (2 nm) could regulate the gene expression of pro-inflammatory cytokines, with hydrophobic GNPs inducing higher levels of *TNF-*α and *IL-6* gene expression in splenocytes (Moyano et al., 2012). However, in another study, hydrophobic ZnO NPs (130 nm) elicited lower expression of ER stress-apoptosis genes compared to pristine ZnO NPs (100 nm) in A549-macrophage co-culture (Liu et al., 2019). Hydrophilic NPs could also alleviate inflammation. For example, three kinds of nanogels (50–60 nm) were synthesized with PEG, poly(sulfobetaine) (PSB), and poly(carboxybetaine) (PCB). PCB nanogels, which is the most hydrophilic, could efficiently alleviate inflammation responses induced by LPS, followed by PSB and PEG nanogels (Li et al., 2018).

*In vivo* experiments demonstrated that hydrophobic polymeric NPs (160 nm) could elicit inflammation in mice. The hydrophobicity of polymeric NPs was positively correlated with neutrophilia and pro-inflammatory cytokine levels (Dailey et al., 2015). Mice treated with hydrophobic pristine-MWCNTs induced inflammatory cell infiltration in the portal region of liver, however, hydrophilic f-MWCNTs induced slight or no inflammation in liver (Jain et al., 2011). PEG decoration can alleviate the inflammation induced by NPs. In HaCaT cells, GNRs (35 × 10 nm) decorated with mercaptohexadecanoic acid upregulated the *IL-1*α and *Serpine1* genes, which corresponded to pro-inflammatory and anti-inflammatory effects, respectively. GNRs decorated with PEG had no impact on these genes (Grabinski et al., 2011). Both pristine and PEG-decorated iron oxide NPs (10 nm) could induce the release of proinflammatory chemokine IL-8 in A549 cells. The secretion level of IL-8 induced by PEG decoration was low compared to that induced by pristine iron oxide NPs (Griffete et al., 2012). In a 28 days repeated dose toxicity study, pristine-MWCNTs caused higher levels of inflammation in lung and liver of mice than PEG-MWCNTs (Zhang et al., 2017). Few-layer graphene (FLG, 2–4 layers, lateral dimension of ~100–200 nm) and FLG-COOH elicited acute and chronic lung inflammation in mice. PEG decoration on FLG could effectively alleviate lung inflammation (Sasidharan et al., 2015). In another *in vivo* study, it was also found that PEG decoration on shell-crosslinked-knedel-like NPs (20–25 nm) could ameliorate the acute inflammation in the lung, and the length of PEG chain was positively correlated with the anti-inflammatory effects (Ibricevic et al., 2013).

### Combinatorial Surface Modifications

Based on a combinatorial MWCNT library, Gao et al found that surface chemistry on MWCNTs could tune the inflammatory response *in vivo* and *in vitro*. The *in vivo* experiments demonstrated that surface chemistry could regulate the IL-1β and TNF-α levels in the lung and liver. The *in vitro* experiments indicated that surface chemistry could steer macrophage recognition from the mannose receptor to the scavenger receptor, resulting in the alleviation of the NF-κB pathway and proinflammatory chemokines (Gao et al., 2011).

## Apoptosis

Apoptotic pathways could be classified into two types: extrinsic and intrinsic pathways. Extrinsic apoptotic pathways could be triggered through the activation of the death receptor superfamily proteins, including CD95 and tumor necrosis factor receptor I. Intrinsic apoptotic pathways are primarily mediated through the mitochondria or the endoplasmic reticulum (Hengartner, 2000; Szegezdi et al., 2006).

There are many approaches to determining apoptosis. For example, the expression level of proapoptotic and antiapoptotic genes can be determined by RT-PCR (Cho et al., 2009b; Schaeublin et al., 2012). Western blotting and immunofluorescence can be used to determine the expression level of proapoptotic and antiapoptotic proteins (Hengartner, 2000). TEM images can also provide apoptosis-related information at the cell level (Elmore, 2007). In this section, we summarize recently published literatures related to the regulation of apoptosis by NPs' surface charge and hydrophobicity (Table 6).

**Table 6:** Apoptosis regulated by NPs' surface chemistry.

**Composition**	**Size**	**Surface chemistry/Zeta potential/LogP**	**Cell line**	**Apoptosis**	**References**
Gold	1.5 nm	Trimethylammoniumethanethiol (TMAT,), mercaptoethanesulfonate (MES), mercaptoethoxyethoxyethanol (MEEE)	HaCaT	(TMAT, MES)>MEEE	Schaeublin et al., 2011
Gold	1–10 nm	Poly(quaternary ammonium), sodium polyacrylate	Human neutrophils	Sodium polyacrylate> poly(quaternary ammonium)	Durocher et al., 2017
Gold	20–25 nm	Cysteamine conjugated cholic acid (DCaC), dicationic cysteamine conjugated deoxycholic acid (DCaDC), dicationic cysteamine conjugated lithocholic acid (DCaLC)	A549	DCaLC> DCaDC>DCaC	Muthukumarasamyvel et al., 2017
Gold nanowires	Diameter: 200 nm	NH_2_ (11.4 mV), COOH (-25.5 mV)	Fibroblast, HeLa	NH_2_ >COOH	Kuo et al., 2007
Graphene	3.5–5 nm	OH, COOH, NH_2_	A549	OH>(COOH, NH_2_)	Xie J. et al., 2019
Graphene	200 nm	PEI (40.4 mV), pristine (−7.36 mV), BSA (−33.3 mV), PEG (−18.3 mV)	J774A.1	PEI> the rest	Luo et al., 2015
Polystyene	110 nm	NH_2_, COOH	THP-1, differentiated THP-1	NH_2_ >COOH	Loos et al., 2014
Polystyene	60 nm	NH_2_ (40.3 mV), COOH (−27.6 mV)	RAW264.7	NH_2_ >COOH	Xia et al., 2006
Polystyene	200 nm	NH_2_, PEG-NH_2_	RAW264.7	NH_2_ >PEG-NH_2_	Lee K. et al., 2011

### Surface Charge

Positively and negatively charged GNPs (1.5 nm) increased the expression of caspase-3 and led to the apoptosis of HaCaT cells, while neutrally charged GNPs induced necrosis instead of apoptosis (Schaeublin et al., 2011). In another study, negatively charged GNPs (1–10 nm) induced apoptosis in freshly isolated human neutrophils, while positively charged GNPs did not elicit apoptosis (Durocher et al., 2017). In fibroblast and HeLa cells, positively charged gold nanowires (diameter: 200 nm) induced greater cytotoxicity than negatively charged counterparts (Kuo et al., 2007).

**Figure 7:**
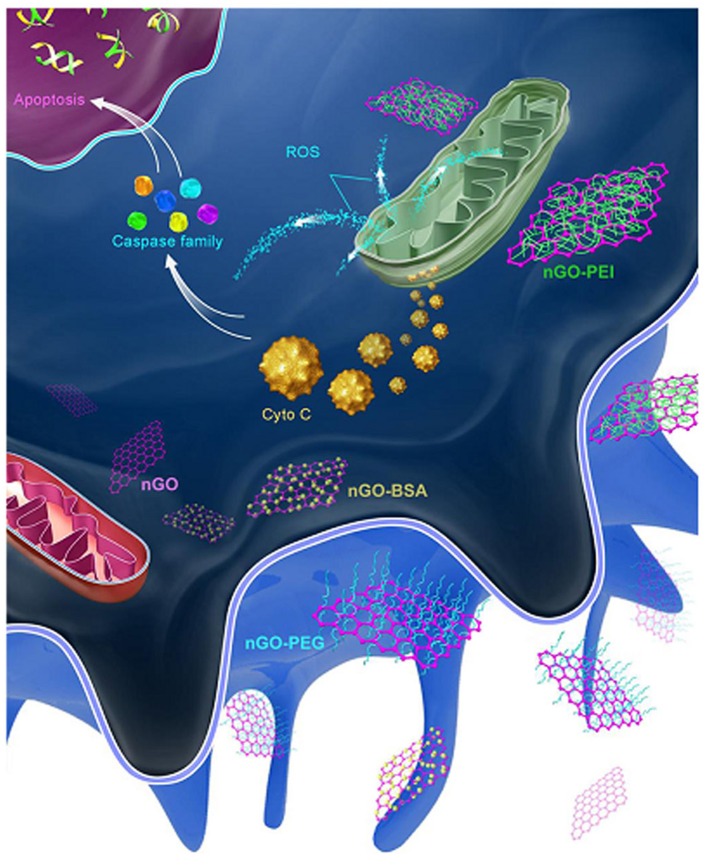
After being phagocytized, PEI-GO was more apt to interact with mitochondria and activate the apoptotic pathway. Reproduced with permission from Luo et al. (2015).

GQDs (3.5–5 nm) with different surface charges can tune apoptosis in A549 cells. Neutrally charged OH-GQDs induced higher levels of apoptosis than did either negatively charged COOH-GQDs or positively charged NH_2_-GQDs (Xie Y. et al., 2019). In J774A.1 macrophages, positively charged PEI-GO NPs induced the highest level of apoptosis, followed by pristine, BSA, and PEG decorated GO NPs, which all had a negative zeta potential (Figure 7) (Luo et al., 2015).

In THP-1 and differentiated THP-1 cells, positively charged NH_2_-PS NPs (110 nm) induced apoptosis, while negatively charged COOH-PS NPs did not induce apoptosis (Loos et al., 2014). In RAW264.7 macrophages, NH_2_-PS NPs (60 nm) was found to elicit apoptosis, but not COOH-PS NPs (Xia et al., 2006). Therefore, on PS NPs, positively charged decorations are more likely to induce apoptosis than are negatively charged decorations.

### Hydrophobicity

The hydrophobicity of GNPs (20–25 nm) could regulate apoptosis in A549 cells, with hydrophobic GNPs inducing higher levels of apoptosis than induced by hydrophilic GNPs (Muthukumarasamyvel et al., 2017). The apoptosis levels in RAW264.7 cells induced by NH_2_- and PEG-NH_2_-PS NPs (200 nm) were compared. PEG decoration significantly reduced apoptosis levels compared to those of NH_2_-PS NPs (200 nm) in the RAW264.7 cells (Lee K. et al., 2011). However, *in vivo* experiments demonstrated that PEG-decorated NPs could also induce apoptosis. For example, PEG- GNPs (4, 100 nm) induced apoptosis in liver tissue after intravenous administration to BALB/c mice (Cho et al., 2009b). In another paper, PEG- GNPs (13 nm) was found to accumulate in liver and induced apoptosis 7 days after injection (Cho et al., 2009a).

## Summary and Perspective

**Figure 8:**
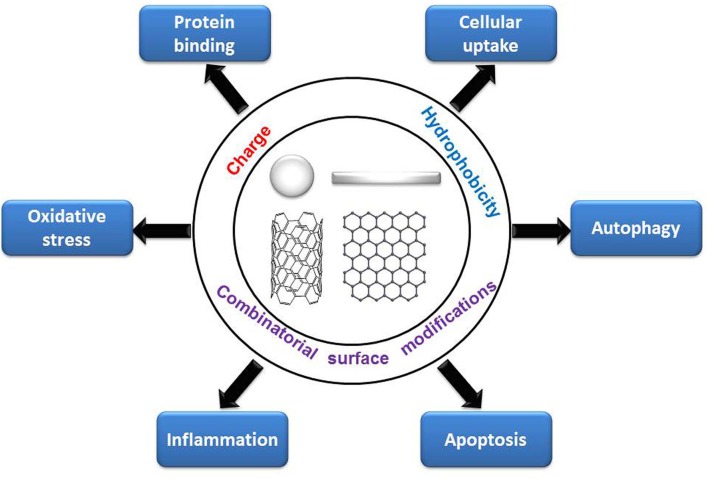
Regulation of cytotoxicity-related bioeffects by surface chemistry.

By summarizing recent literature, we found that the decoration of PEG, ligands of different charge and hydrophobicity, and combinatorial surface decoration could tune various cytotoxicity-related bioeffects. Generally, positively charged and hydrophobic NPs are more likely to be internalized by cells and induce oxidative stress, autophagy, and apoptosis than are negatively charged or hydrophilic NPs, and negatively charged NPs are more likely to adsorb proteins than are positively charged NPs. PEG decoration on NPs can alleviate these cytotoxicity-related bioeffects (Figure 8). Adding combinatorial surface decoration was proven to be an effective strategy to reveal the impact of various types of surface chemistry on bioeffects.

In addition to the bio-effect levels, molecular pathways can also be regulated by surface chemistry, especially for differently charged, hydrophobic, and combinatorial surface decorations. In our study, we found that oxidative stress induced by positively charged and hydrophobic GNPs is endocytosis-dependent. The endocytosis of positively charged and hydrophobic GNPs elicited oxidative stress through different signaling pathways, including elevated calcium level, activation of mitochondria and NADPH oxidase. Moreover, oxidative stress is considered to be an important upstream mechanism that positively regulate autophagy, inflammation, and apoptosis (Circu and Aw, 2010; Reuter et al., 2010; Lee J. et al., 2011). Given that surface charge and hydrophobicity are non-specific interactions between NPs and biomolecules (Kim et al., 2013; Nam et al., 2013), binding of specific key proteins may not be the main mechanism for surface charge or hydrophobicity induced cytotoxicity response. From the published papers, we can see that the mechanism is complicated, from affecting the protein binding, the internalization pathways and subcellular localization of NPs to the interaction with the cell membrane, lipids, proteins, and DNA, and the induction of oxidative stress as well.

For NPs of different compositions, sizes and shapes, the rules by which surface chemistry regulates bioeffects are sometimes inconsistent. Moreover, the surface chemistry modulation of bioeffects sometimes varies by cell line. Therefore, caution should be exercised when extrapolating conclusions obtained from a certain sized, shaped, or composited NP and/or a cell line.

## Author Contributions

HS and SZ designed this work of review and revised the manuscript. HS, CJ, LW, and XB performed the literature search of the databases. HS, CJ, LW, XB, and SZ wrote the manuscript. All authors approved the paper for publication.

### Conflict of Interest

The authors declare that the research was conducted in the absence of any commercial or financial relationships that could be construed as a potential conflict of interest.
